# Nardosinone Alleviates Parkinson's Disease Symptoms in Mice by Regulating Dopamine D2 Receptor

**DOI:** 10.1155/2021/6686965

**Published:** 2021-08-13

**Authors:** Li-hua Bian, Zi-wei Yao, Cheng-bowen Zhao, Qiu-yu Li, Jin-li Shi, Jian-you Guo

**Affiliations:** ^1^School of Chinese Materia Medica, Beijing University of Chinese Medicine, 11A North Third Ring East Road, Chaoyang District, Beijing 100029, China; ^2^CAS Key Laboratory of Mental Health, Institute of Psychology, Chinese Academy of Sciences, 4A Datun Road, Chaoyang District, Beijing 100101, China

## Abstract

Nardostachyos Radix et Rhizoma (nardostachys) is the root and rhizome of *Nardostachys jatamansi* DC. Recent studies have shown that nardostachys may exert an anti-PD effect. In this study, the UHPLC-LTQ-Orbitrap-MS method was used to analyze the brain components of nardostachys in rats. Based on the results of UHPLC-LTQ-Orbitrap-MS analysis, nardosinone was identified to be the most effective anti-PD compound in nardostachys. To further verify this inference, a mouse PD model was established and the effect of nardosinone on PD mice was determined using classic behavioral tests. The results showed that nardosinone was indeed effective for relieving PD symptoms in mice. Moreover, network pharmacology analysis was used to elucidate the mechanism underlying the anti-PD effect of nardosinone. Dopamine receptor D2 (DRD2) was identified as the key target of nardosinone-PD interaction network, which was further verified by molecular docking and Western blotting. The results demonstrated that nardosinone and DRD2 could interact with each other. Furthermore, the expression level of DRD2 was decreased in the brain tissue of PD mice, and nardosinone could restore its expression to a certain extent. In conclusion, our findings suggest that nardosinone may reduce the motor and cognitive symptoms in the animal PD model by regulating DRD2 expression.

## 1. Introduction

Parkinson's disease (PD) is a common disease among middle-aged and elderly people and is the second most common neurodegenerative disease after Alzheimer's disease [1, 2]. The main pathological features of PD are the progressive, extensive degeneration and deletion of dopamine (DA) neurons in the dense substantia nigra as well as the high expression of *α*-synuclein (a major constituent of Lewy bodies) [3]. An extensive loss of dopaminergic neurons can decrease the expression levels of DA in the striatum, which leads to a series of clinical symptoms such as resting tremor, abnormal posture gait, muscular dyskinesia, hyposmia, constipation, depression, and so on [4]. This may severely affect patients' physical health and quality of life.

The etiology of PD remains largely unknown [5]. Epidemiological studies indicate that a number of factors may increase the risk of PD development [6], including genetic susceptibility, oxidative stress, mitochondrial defects, neurological inflammation, neurostimulant toxicity, and accumulation of abnormal proteins [7–9]. These factors can lead to the occurrence of apoptosis; thus, in the course of the disease, the excessive apoptosis of dopaminergic neurons is the central link to neurodegenerative diseases. PD remains incurable [10] because the currently available therapies are not able to prevent or reverse the progression of the disease [11]. At present, the main drug used for the treatment of PD is levodopa, which can increase the level of DA in the brain and/or mimic the effect of DA. However, after its long-term use, some side effects such as “switching phenomenon,” end-stage phenomenon, and mental symptoms [12, 13] may occur, which hinder its application for PD treatment. Therefore, it is of utmost urgency to develop a new drug that can prevent or delay the onset of PD.

Nardostachyos Radix et Rhizoma (nardostachys) is the root and rhizome of *Nardostachys jatamansi* DC., which has the effects of regulating Qi and relieving pain, opening depression, and waking up the spleen [14]. Previous studies have suggested that nardostachys exhibits certain pharmacological activity on the nervous system [15, 16] and can relieve PD symptoms in rats [17]. If the active drugs of the central nervous system do not pass through the blood-brain barrier, it will be difficult for them to exert substantial pharmacological effects [18]. Therefore, anti-PD drugs need to penetrate the blood-brain barrier to exert their efficacy. To clarify the anti-PD active ingredients in nardostachys, we analyzed the brain components of PD rats after treatment with nardostachys. A total of 5 compounds were detected in the brain tissues of PD rats, among which nardosinone caught our attention.

Nardosinone is a major active component of nardostachys, and its content is as high as 2.9% [19]. Nardosinone has been shown to promote the proliferation of mouse embryonic neural stem cells [20] as well as the synaptic growth of PC12D cells [21]. It is speculated that nardosinone has certain pharmacological activities on the nervous system, and it is considered to have anti-PD effects. More importantly, the acute toxicity studies of nardosinone have shown that its toxicity is relatively low [22]. Therefore, nardosinone may be an ideal drug for the treatment of neurodegenerative diseases, especially PD.

Rotenone is a natural organic insecticide that can easily penetrate the blood-brain barrier. It has a strong inhibitory effect on the activity of mitochondrial respiratory chain complex I in brain tissues [23] and selectively causes neural degeneration in the nigrostriatal DA system [24, 25]. Lewy bodies typically appear in the dopaminergic neurons of rats exposed to rotenone for a long period of time [26]. Besides, the rats and mice exposed to rotenone also exhibit PD-like clinical symptoms such as sluggishness and stiffness [27]. At present, the subcutaneous injection of rotenone on the neck and back has become a common method for establishing PD models. Therefore, in this study, rotenone was used to induce PD in mice.

Network pharmacology is a new discipline that reveals the regulatory network of drugs on the body at the system level. It can predict the mechanism of drug action by constructing a complex network relationship among “drugs, active ingredients, targets, and diseases,” especially for the mechanistic prediction of multitarget drugs such as traditional Chinese medicine. This web-based drug discovery has the advantages of economy, convenience, and reliability.

In this study, rotenone was used to establish a mouse PD model, and the anti-PD efficacy of nardostachys was verified through behavioral experiments (e.g., cognitive training). The molecular mechanism underlying the anti-PC effect of nardosinone was elucidated by network pharmacology-based analysis. To verify the accuracy of network pharmacology-based prediction, molecular docking was carried out. Western blotting was then performed to detect the protein expression of dopamine receptor D2 (DRD2).

## 2. Materials and Methods

### 2.1. Sample Preparation

Nardostachys samples were prepared by natural drying and fine pulverization. The powder (−100 mesh) was weighed to 10 g and extracted with 300 mL of 80% ethanol (ultrasonic extraction for 60, 45, 45 min). The extracts were then filtered by six layers of gauze and dried under reduced pressure at <40°C.

### 2.2. Animals

#### 2.2.1. Animals Used for the Detection of Brain Components

A total of 24 male Wistar rats (weighing 180–210 g) were purchased from Beijing Charles River Laboratory Animal Technology Co., Ltd. On the first day of arrival, the rats were individually housed in a controlled environment (21∼25°C; 55 ± 10% relative humidity) and maintained in a 12:12 h light/dark cycle (light on, 07:00–19:00 h). They were also allowed to eat and drink freely. All rats were habituated to the laboratory environment for 1 week and gently handled daily.

#### 2.2.2. Animals Used for Behavioral Testing and Western Blotting

C57BL/6N mice (weighing 19–20 g) were obtained from Beijing Charles River Laboratory Animal Technology Co., Ltd. All mice were maintained under standard environmental conditions: 22 ± 2°C, ∼46% humidity, and a 12:12 h light/dark cycle (light on, 7:00–19:00 h). The mice were given *ad libitum* access to food and water. All experiments were performed in a quiet room under dim red light between 8:00 AM and 12:00 PM. Every effort was made to minimize the number of animals used and their suffering. The experimental procedures were approved by the Animal Care and Use Committee of the Institute of Psychology of the Chinese Academy of Sciences and were in compliance with the National Institutes of Health Guide.

### 2.3. Detection of Brain Tissue Composition in Rats

#### 2.3.1. Mass Spectrometer Detection

The Q-Exactive mass spectrometer and ultra-high performance liquid chromatography (UHPLC) system (Vanquish) were equipped with heat spray ion source (HESI) for sample analysis. Positive ion detection mode, ion source temperature of 400°C, spray voltage of 3.5 kV, S-lens RF voltage of 21 V, capillary temperature of 320°C, sheath gas and auxiliary gas of high pure nitrogen (purity >99.99%), sheath gas flow rate of 35 arb, and auxiliary gas flow rate of 10 arb were employed. Mass spectral data were collected in a full-scan range of 150–1500 m/z and data-dependent acquisition second-level mass spectrometry (ddMS2). The full-scan resolution was 70,000, MS/MS resolution was 13500, and the collision mode was high-energy collision ionization (HCD) with normalized collisional energies (NCEs) of 30% and 50%.

#### 2.3.2. Chromatographic Conditions

Chromatographic column UHPLC BEH C18 (2.1 mm × 50 mm, 1.7 *μ*m) was used at a flow rate of 0.3 mL/min. The column temperature was set at 25°C, and the injection volume was 3 *μ*L. The mobile phase contained a mixture of ultrapure water with 0.1% formic acid (A) and acetonitrile (B). The following gradient program was used for brain tissue: 0–10 min, 95% (B); 10–50 min, 45–5% (B); 50–55 min, 5–95% (B) followed by a reequilibration step in 95% (B) for 5 min. The following gradient program was used for cerebrospinal fluid: 0–30 min, 95% (B); 30–90 min, 95–35% (B); 90–95 min, 5–95% (B) followed by a reequilibration step in 95% (B) for 5 min.

#### 2.3.3. Sample Collection

Wistar rats were taken, 4 in the blank group and 20 in the five experimental groups (*n* = 4 per group). All rats were fasted for 12 h (free drinking water) before the experiments, and nardostachys was administered in accordance with the adult raw medicine dosage of intragastric administration.

An appropriate amount of dry extract was weighted, ground evenly, and mixed with 1% sodium carboxymethyl cellulose. The rats in blank control group were given 1% sodium carboxymethyl cellulose solution.

The rats were given IG for 7 times consecutively at a time interval of 12 h. After the last administration, the abdominal aorta was extracted at 0.5, 1, 1.5, 2, and 2.5 h, respectively, by placing in a disposable vacuum vessel with heparin sodium. The samples at each time point were stored in a refrigerator (−20°C) until further use.

#### 2.3.4. Pretreatment

The brain tissue samples were added with normal saline at a ratio of 1 : 3 (W : V), homogenized by an ultrasonic crusher, and then centrifuged at 13000 R/min for 30 min at 4°C. A C18 SPE solid phase extraction column was used. The column was activated with 3 ml methanol, and then 3 ml of deionized water was added to balance the SPE column. Approximately 2 ml of tissue samples was put into the activated solid phase extraction column, washed with 4 ml deionized water, and then eluted with 3 ml methanol. The collected methanol eluent was dried with N_2_ at room temperature. The residue was redissolved with 200 *μ*l methanol, whirled for 3 min, and then centrifuged at 13000 R/min for 15 min at 4°C. Finally, the supernatant was collected for sample analysis.

The cerebrospinal fluid samples (400 *μ*l) were precisely aspirated, followed by the addition of methanol for 4 times. After 3 min of vortexing, the mixture was centrifuged at 13000 R/min for 15 min at 4°C. The supernatant was then collected and dried with N_2_. The residue was redissolved with 200 *μ*l methanol, whirled for 3 min, and centrifuged at 13000 R/min for 15 min at 4°C. Lastly, the supernatant was collected and subjected to further analysis.

#### 2.3.5. Data Processing

Xcalibur 4.0 and Compound Discovered software were used to analyze the chemical composition of nardostachys in the brain tissues. Image J software was used to transform the protein band image into gray image, and the gray value of each band was then analyzed. The gray value of DRD2 protein was divided by that of GAPDH protein for normalization.

### 2.4. Rotenone-Induced PD Model

C57BL/6N mice were randomly assigned to three groups (*n* = 8 per group): sham operation group (Control group), rotenone injection group (ROT group), and rotenone modeling + nardosinone group (ROT + NAR group). All mice were fed adaptively for 7 days. From day 8 to day 21, the mice in control group were injected subcutaneously with normal saline (3 ml/kg body weight) on the back of the neck. Meanwhile, those in ROT group were subcutaneously injected with 3 mg/kg body weight of rotenone (95% purity; Aladin company, China), sunflower oil (W18A8L41924; Shanghai yuanye Bio-Technology Co., Ltd, China) solution on the back of the neck. After injection, 8 mice with dyskinesia were selected for the subsequent experiments. On days 21–25, the mice in ROT + NAR group received an intraperitoneal injection of 80 *μ*g/g nardosinone (98% purity; 200492–170108; Yongjian Medicine, China) in 2% sodium carboxymethylcellulose solution for 5 consecutive days. The remaining groups were injected with 2% sodium carboxymethylcellulose solution.

### 2.5. Behavioral Test

#### 2.5.1. Open Field Test

After acclimatization for 30 min, the mice were placed in an open field (40 cm × 40 cm). Starting from the edge, they were allowed to move freely for 5 min, and their total distance traveled was recorded.

#### 2.5.2. Shuttle Box

A shuttle box was used to examine the cognitive ability of PD mice. It consisted of two chambers that were separated by a guillotine door. There was an intermittent electric shock on the floor of the dark compartment. The mice were placed into the left chamber and back to the hole. When they entered the dark module, the door was closed. The alternating current was set at 0.2 mA, training interval was random, and average time was 15 s. Each mouse was trained from 30 times. Twenty-four hours later, the experiment was repeated, and the total distance traveled and total movement time were recorded.

### 2.6. Data Preparation

#### 2.6.1. Therapeutic Target Database Searching

The known therapeutic targets of PD drugs were acquired from the Therapeutic Target Database (TTD; https://db.idrblab.org/ttd/) [28] and Pharmacogenomics Knowledge Base (PharmGKB; https://www.pharmgkb.org/) [29], with “Parkinson” as the search term. By integrating the obtained target protein information and eliminating the repeated targets in the search results, the known targets responsible for the pathogenesis of PD were obtained. In the UniProt database (https://www.uniprot.org/) [30], the species was adjusted to “Homo sapiens,” while drug targets, protein names, and gene names were uniformly standardized.

#### 2.6.2. Prediction of the Component Targets for Nardosinone

The traditional Chinese medicine systems pharmacology database and analysis platform (TCMSP; http://lsp.nwsuaf.edu.cn/tcmsp.php) [31], Swiss Target Prediction database (http://www.swisstargetprediction.ch/) [32], and PharmMapper database (PharmMapper http://www.lilab-ecust.cn/pharmmapper/) [33] were used to predict the candidate targets of nardosinone, with the species set as “Homo sapiens.” Meanwhile, UniProt database (https://www.uniprot.org) was used to search for protein target IDs and gene IDs, organize the target information of nardosinone targets, and remove duplicates.

#### 2.6.3. Protein-Protein Interaction (PPI) Network Analysis

STRING platform (https://string-db.org/) was used to construct an interaction network among the target proteins. The protein type was set as “Homo sapiens,” the minimum interaction threshold was adjusted to “Medium confidence,” and other parameters were kept at default values. Cytoscape 3.7.1 software (http://www.cytoscape.org) was used to visualize the PPI network between PD-related genes and nardosinone-target encoding genes.

#### 2.6.4. Bioinformatics Analysis of Nardosinone-PD Targets

To assess the biological significance of specific genes or proteins for nardosinone-PD targets, gene ontology (GO) and Kyoto Encyclopedia of Genes and Genomes (KEGG) pathway enrichment analyses were performed using DAVID (https://david.ncifcrf.gov/) database.

### 2.7. Molecular Docking

The structure of DRD2 was downloaded from RCSB protein database (http://www.rcsb.org/pdb), and the receptor protein was analyzed using PyMOL software. The 3D chemical structure of nardosinone, with an optimized energy minimization, was constructed using Chembiodraw 3D, which was saved in mol2 format. Autodock tools were used to set flexible residues, appropriate box centers, and box lattice parameters, to contain the active pocket sites that may bind to small-molecule ligands. Molecular docking of the receptor proteins and small-molecule ligands was carried out using Autodock Vina. PyMOL software was used to draw the best scoring conformation.

### 2.8. Western Blotting

Antibody against DRD2 was obtained from Beijing BIOSS Biotechnology Co., Ltd. (Beijing, China). The membrane was incubated with DRD2 antibody and GAPDH (Proteintech, 1 : 500) overnight at 4°C. After washing with PBS for 3 times, the membrane was incubated with goat anti-rabbit HRP secondary antibody (Shanghai Hua'an Co., Ltd., Shanghai, China). The film was then scanned with Gel Imaging system (Tanon Science & Technology Co., Ltd., Shanghai, China). Protein bands were quantified using ImageJ software. GAPDH was used as the standard reference.

### 2.9. Statistical Analysis

All analyses were carried out using GraphPad Prism Version 5. Behavioral test data were expressed as mean ± standard error of mean (SEM) of at least three independent experiments. Statistical difference between groups was determined using one-way analysis of variance (ANOVA). A *P* value of <0.05 was considered statistically significant.

## 3. Results

### 3.1. Identification of Compounds in the Brain Tissue of Rats Treated with Nardostachys Extract

**Figure 1:**
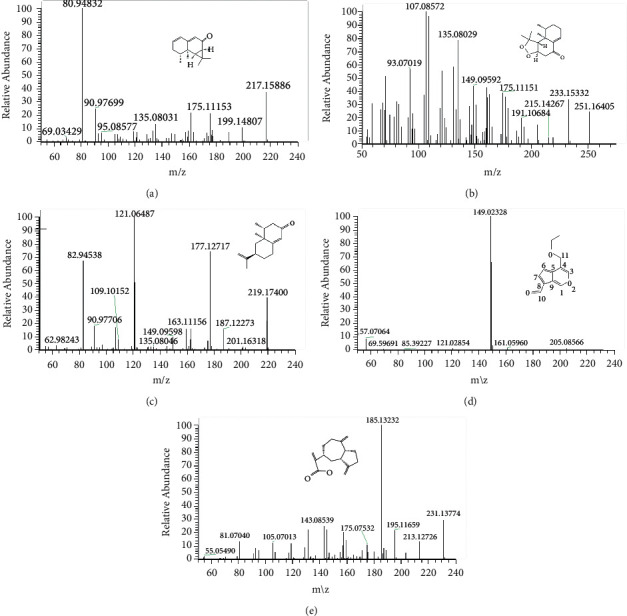
UHPLC-LTQ-Orbitrap-MS spectra of nardostachys extract in rat brain tissue (positive ions). Kanshone H (a), nardosinone (b), nootkatone (c), 11-ethoxyviburtinal (d), and dehydrocostus lactone (e).

The pretreated tissue samples were collected for injection analysis according to the retention time of each chemical component, high-resolution accurate molecular weight, MS2 level fragment information, together with the fragmentation rule of standard substance, fragment information, and Online database information (https://pubchem.ncbi.nlm.nih.gov/, http://www.hmdb.ca/).The results showed that five compounds, namely, kanshone H, nardosinone, nootkatone, 11-ethoxyviburtinal, and dehydrocostus lactone, were characterized in the brain tissues pretreated with the ethanolic extract (80%) of nardostachys (Table 1; Figure 1).

### 3.2. Nardosinone Reverses Rotenone-Induced Motor and Cognitive Impairment in Mice

**Figure 2:**
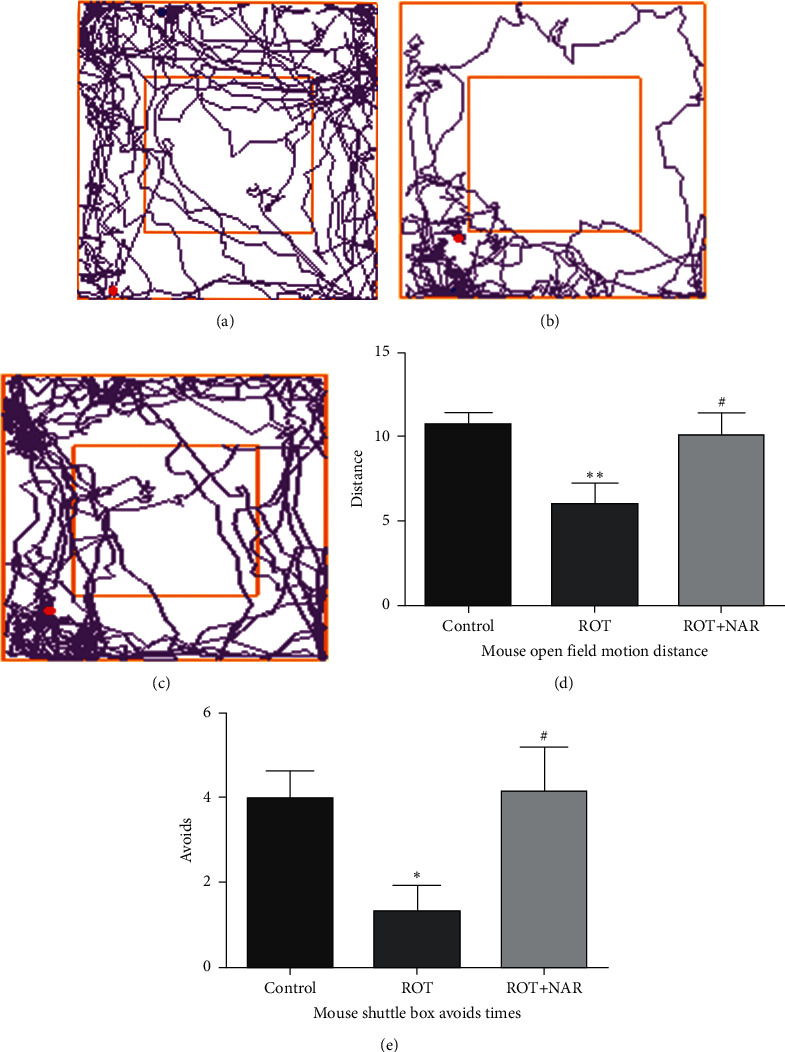
Nardosinone reverses rotenone-induced motor and cognitive impairment in mice. The open field motion trajectories of control group (a), ROT (subcutaneously injected with 3 *μ*g/g/day body weight of rotenone) group (b), and ROT + NAR (intraperitoneal injection of 80 *μ*g/g/day nardosinone) group (c). (d) Open field movement distance of mice in each group. (e) Number of the active avoidance of shuttle box in each group (results are expressed as $\bar{x}\pm s$ (*n* = 6); ^*∗*^*P* < 0.05 and ^*∗∗*^*P* < 0.01 versus control group; ^#^*P* < 0.05 versus ROT group).

The open field track plots of mice in sham operation group (control), rotenone group (ROT), and rotenone + nardosinone group (ROT + NAR) are shown in Figure 2(d), while the total open field movement distances of mice in the three groups are presented in Figure 2(e). Compared with the PD model group, the open field movement distance of mice in the NAR group was significantly increased (*P* < 0.05). These results indicated that nardosinone could remarkably improve the dyskinesia of PD mice induced by rotenone.

### 3.3. Identification of PD-Related Targets

**Figure 3:**
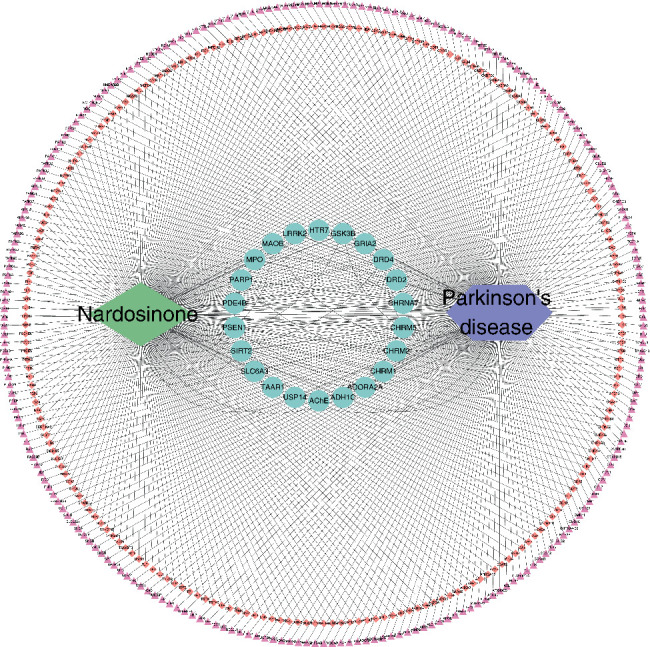
Nardosinone-PD-target network (the purple triangles represent targets for PD, the pink diamonds represent the targets of nardosinone, the green circle represents the intersection target of nardosinone and PD).

A total of 80, 183, and 6 PD-related targets were obtained from TTD, OMIM, and PharmGKB platforms, respectively. After merging the targets predicted by the three platforms and deleting duplicates, a total of 259 PD-related targets were identified (Figure 3).

### 3.4. Identification of Nardosinone Targets

A total of 9, 99, and 152 nardosinone targets were retrieved from TCSMP, Swiss Target Prediction, and PharmMapper databases, respectively. After merging the targets predicted by the three databases and removing duplicates, 238 nardosinone targets were identified (Figure 3).

### 3.5. PPI Network of Anti-PD Targets for Nardosinone

**Figure 4:**
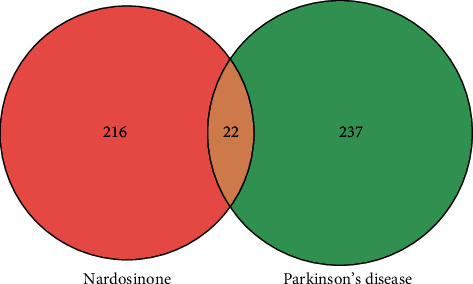
Wayne diagram of nardosinone targets and PD targets.

**Figure 5:**
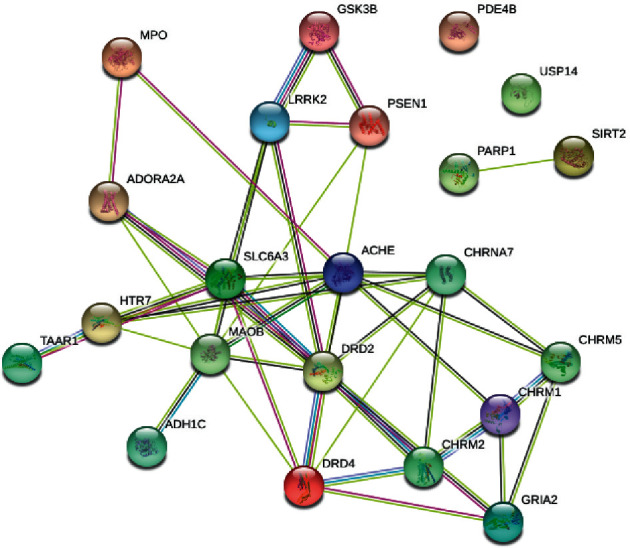
PPI network of nardosinone-PD targets.

The intersection targets of nardosinone and PD were screened, and there are 22 common targets between nardosinone and PD (Figure 4). All interaction targets were imported into the STRING platform for PPI network analysis (Figure 5). The topology analysis in Cytoscape was used to process PPI data and measure node centrality, including degree centrality (DC), closeness centrality (CC), and betweenness centrality (BC). It was found that DRD2 exhibited the highest DC, CC, and BC values. This indicates that DRD2 is in the most important position in the network.

### 3.6. GO and KEEG Pathway Enrichment by Nardosinone for Potential PD Targets

**Figure 6:**
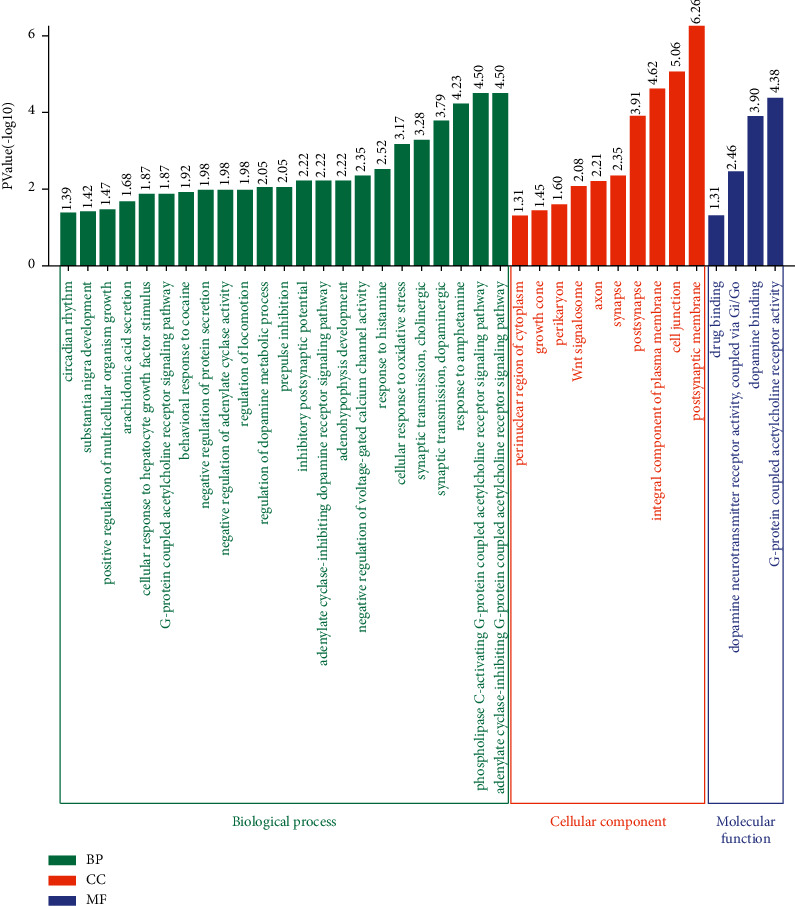
GO enrichment analysis of nardosinone-PD targets.

GO functional annotation showed that the most relevant GO term was “synaptic transmission, cholinergic.” The key targets were mainly involved in the biological process (BP) of the “conduction of various neurotransmitters,” “the development of neurons and the nervous system,” cellular component (CC) of “cell synapses,” and has molecular function (MF) of “G protein coupling acetylcholine receptor” and “dopamine binding cellular” (Figure 6).

**Figure 7:**
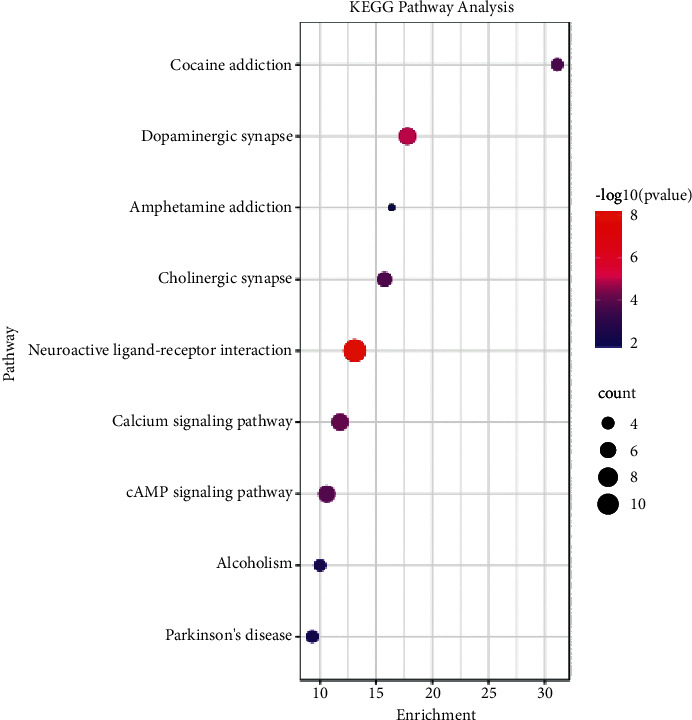
KEGG enrichment analysis of nardosinone-PD targets.

KEGG pathway analysis revealed that there were eight pathways associated with the anti-PD effects of nardosinone. These included the neuroactive ligand-receptor interaction pathway, dopaminergic synapse, calcium signaling pathway, cAMP signaling pathway, cholinergic synaptic pathway, cocaine addiction, alcoholism, Parkinson's disease pathway, amphetamine addiction, nicotine addiction, tyrosine metabolism, and regulation of actin cytoskeleton (Figure 7). It was found that DRD2 appeared most frequently in the enrichment results. Combined with PPI network and topology analyses, the mechanism underlying the anti-PD effects of nardosinone was closely related to DRD2.

### 3.7. Molecular Docking

**Figure 8:**
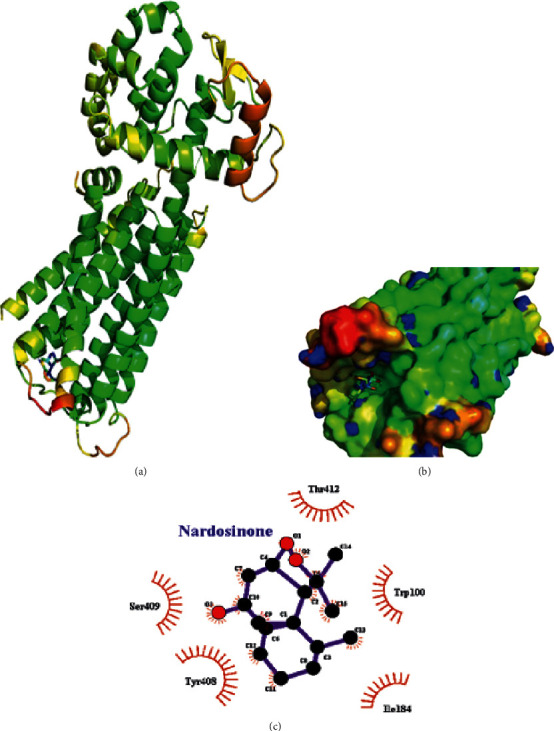
The docking results and interaction analysis of nardosinone and DRD2. (a) The optimal idea of docking nardosinone with DRD2; (b) the local method diagram of the docking part of nardosinone and DRD2; (c) the interaction force between nardosinone and DRD2.

The results of molecular docking indicated that nardosinone could bind to DRD2 receptor in the capsule cavity, with a low binding energy of −6.6. From the analysis of the interaction between DRD2 and nardosinone, it was found that DRD2 could bind to nardosinone via threonine, tryptophan, lysine, isoleucine, serine, etc. (Figure 8).

### 3.8. Expression of DRD2

**Figure 9:**
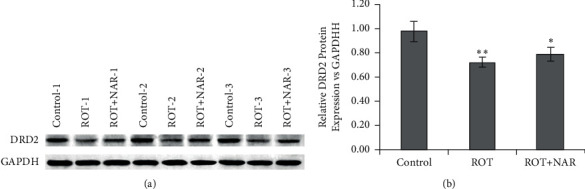
Expression of DRD2: (a) Western blot results; (b) histogram of gray value analysis. ^*∗*^*P* < 0.05 and ^*∗∗*^*P* < 0.01 versus control group.

To further verify the network pharmacology-based prediction results, the expression levels of DRD2 were measured and compared among the control, ROT, and ROT + NAR groups (Figure 9). The results showed that the expression level of DRD2 was decreased in the brain tissue of ROT mice when compared to the control mice. However, treatment with nardosinone significantly upregulated the expression level of DRD2 in the brain tissue of rotenone-exposed mice.

## 4. Discussion

After intragastric administration of nardostachys extract, the brain tissue compositions of rats were analyzed by UHPLC-LTQ-Orbitrap-MS. A total of 5 compounds were identified, namely, kanshone H, nardosinone, nootkatone, 11-ethoxyviburtinal, and dehydrocostus lactone. A literature search was conducted on these chemical components. Dehydrocostus lactone has anticancer [34] and antioxidative stress [35] effects; nootkatone has anti-inflammatory [36], antitumor [37], and anti-Alzheimer's [38] effects; nardosinone is a major active component of nardostachys. It has antianxiety, antidepressant, and neuroprotective effects. Wang [39] conducted an *in vitro* study on the anti-PD effects of nardosinone, and the results showed that nardosinone had significant protective effects on 6-OHDA-induced SH-SY5Y cells. Based on this, it can be inferred that, among these 5 active ingredients, nardosinone has the greatest anti-parkinsonian potential. In addition, rotenone-induced mouse PD model was used to verify the anti-PD efficacy of nardosinone. The results showed that nardosinone could improve the two main PD symptoms (e.g., motor and cognitive abilities) of rotenone-induced PD mice. Furthermore, the mechanism of nardosinone against PD was evaluated by network pharmacology-based analysis. It was found that nardosinone interacted with 15 targets of PD; the key targets were mainly involved in biological processes such as the conduction of various neurotransmitters, the development of neurons, and the nervous system. The major pathways associated with the anti-PD effect of nardosinone were the interaction of the neuroactive ligand-receptor interaction pathway, calcium signaling pathway, and cAMP signaling pathway.

6-OHDA, MPTP, and rotenone are the neurotoxins commonly used for constructing animal PD models [34]. However, 6-OHDA requires intracerebral injection, is difficult to be partially damaged, and does not simulate early PD, and the success rate of the model is low [40]. The MPTP model has various administration routes, high reliability, and good reproducibility, but the sensitivity of this model varies greatly [41]. For the PD model established by rotenone, the animal behavior showed bow back and low activity; some developed into ankylosis and tremor; the substantia nigra and striatum were positive for ubiquitin and *α*-synuclein staining; the formation of Lewy body-like inclusion bodies was observed [24], which was more effective in mimicking the pathological characteristics of PD patients. Therefore, the rotenone-induced PD model was selected for this study.

Network pharmacology can be employed to predict the interaction among multiple drug targets. It is a rapid and accurate method for target-based drug discovery and has been used to elucidate the mechanism of traditional Chinese medicine [42, 43]. In this study, network pharmacology-based analysis, combined with animal behavior experiments, was performed to determine the efficacy and mechanism of nardosinone. Additionally, molecular docking was carried out to verify the accuracy of the prediction targets. Combined with the results of Western blot analysis, it was found that nardosinone could improve the symptoms of PD in mice by upregulating DRD2 expression.

**Figure 10:**
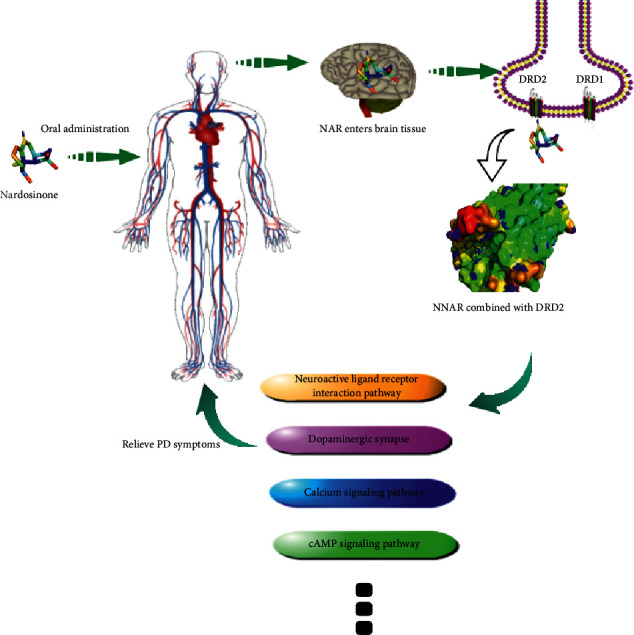
Hypothesis for the mechanism of action of nardosinone in relieving Parkinson's symptoms. (Through the circulation of the human body, nardosinone passes through the blood-brain barrier, enters the brain and binds with DRD2 receptor, and plays a role by regulating multiple signaling pathways.)

DA plays an important role in mediating the pathogenesis and treatment of PD. There are five types of dopamine receptors (DRD), namely, DRD 1-5 [44]. Among them, DRD2 has attracted considerable attention since its agonists can improve the motor symptoms of PD patients without exhibiting the side effects of movement disorders and mental illness. PD patients are often prescribed high-selective DRD2 agonists to control the disease [46–48]. Previous research has shown that DRD2 gene expression is significantly associated with PD [49–51]. The docking results of nardosinone and DRD2 showed that they strongly interacted with each other. Previous research has shown that DRD2 can alleviate PD and prevent the activation of inflammatory responses through *β*-arrestin-2-dependent pathway [52]. The Western blot results further confirmed that the expression of DRD2 was upregulated by nardosinone. Therefore, it is speculated that nardosinone can serve as a DRD2 agonist to alleviate PD by regulating multiple signaling pathways (mechanism hypothesis shown in Figure 10).

## 5. Conclusions

In this study, we verified the anti-Parkinson effect of nardosinone, an effective component of Chinese traditional medicine, and analyzed its mechanism of action through a network pharmacology approach. DDR2 was identified as the key target of nardosinone-PD interaction network, which was further verified by molecular docking and Western blotting. These findings provide the basis for the development of nardosinone as an effective anti-Parkinson drug and also provide a new method for screening active components in Parkinson's medicine.

## Figures and Tables

**Table 1:** UHPLC-LTQ-Orbitrap-MS data for the five compounds of nardostachys extract in rat brain tissue (positive ions).

No.	tR/min	Molecular formula	Theoretical value	Actual value	Error	MS fragment ion information	Conjectural compound
1	25.99	C_15_H_20_O	217.15869	217.15886	0.774	95.08577, 119.08554, 133.10089, 161.09587, 175.11153	Kanshone H
2	25.92	C_15_H_22_O_3_	251.16517	251.16405	−0.482	107.08572, 121.10128, 135.08029, 149.09592, 175.11151, 233.15332	Nardosinone
3	29.66	C_15_H_22_O	219.17434	219.17400	−1.560	109.10152, 159.11667, 163.11156	Nootkatone
4	38.42	C_12_H_12_O_3_	205.08592	205.08566	−1.272	105.07053, 133.06528	11-Ethoxyviburtinal
5	31.53	C_15_H_18_O_2_	231.13795	231.13744	−2.234	81.07040, 93.07020, 105.07013, 119.08569, 143.08539, 157.10104, 185.13232, 195.11695, 213.12726	Dehydrocostus lactone

## Data Availability

The datasets generated and/or analyzed in the present study are included within the manuscript.
